# Thin Graphene–Nanotube Films for Electronic and Photovoltaic Devices: DFTB Modeling

**DOI:** 10.3390/membranes10110341

**Published:** 2020-11-13

**Authors:** Dmitry A. Kolosov, Vadim V. Mitrofanov, Michael M. Slepchenkov, Olga E. Glukhova

**Affiliations:** 1 Department of Physics, Saratov State University, Astrakhanskaya street 83, 410012 Saratov, Russia; kolosovda@bk.ru (D.A.K.); mitrofanovvadimv@gmail.com (V.V.M.); slepchenkovm@mail.ru (M.M.S.); 2 Laboratory of Biomedical Nanotechnology, I.M. Sechenov First Moscow State Medical University, Trubetskaya street 8-2, 119991 Moscow, Russia

**Keywords:** graphene, carbon nanotubes, composite films, volt-ampere characteristics, photocurrent, absorption coefficient

## Abstract

Supercell atomic models of composite films on the basis of graphene and single-wall carbon nanotubes (SWCNTs) with an irregular arrangement of SWCNTs were built. It is revealed that composite films of this type have a semiconducting type of conductivity and are characterized by the presence of an energy gap of 0.43–0.73 eV. It was found that the absorption spectrum of composite films contained specific peaks in a wide range of visible and infrared (IR) wavelengths. On the basis of calculated composite films volt-ampere characteristics (VAC), the dependence of the current flowing through the films on the distance between the nanotubes was identified. For the investigated composites, spectral dependences of the photocurrent were calculated. It was shown that depending on the distance between nanotubes, the maximum photocurrent might shift from the IR to the optical range.

## 1. Introduction

Over the past decade, composite materials based on single-walled carbon nanotubes (SWCNTs) and graphene monolayers have attracted close attention of many researchers [1,2,3,4,5,6,7,8,9]. Technologies for the synthesis of ultrathin all-carbon heterostructures based on SWCNTs and graphene connected by covalent bonds [9,10,11,12] or by van der Waals forces [13,14,15,16] have already been developed and are being applied. Notable advances have also been made in the synthesis of a hybrid graphene/SWCNT structure with individual microscopic morphology [10,11], which opens up the possibility of flexible turning of the physical properties of graphene/SWCNT composites. The first experimental studies on the properties of hybrid graphene/SWCNT composites showed that the intercalation of horizontally oriented SWCNTs between graphene sheets enhances the mechanical strength [10] and improves the on/off current ratio of graphene [17]. The high stretchability and optical conductivity of graphene/SWCNT composite films make them promising candidates for flexible optoelectronics [15,18,19]. The polymerized SWCNT/graphene hybrid layered structures have excellent electrochemical properties, namely, a high specific capacity (~630 mA·h·g^−1^), outstanding power capability (~390 mA·g^−1^), and cyclability of more than 1000 cycles with high Coulombic efficiency [20]. An important step towards the possible use of graphene/SWCNT composites in electronics was the establishment of the charge transfer in junctions formed by covalently bonded graphene and SWCNTs [21].

Due to the existing electronic coupling between two sp^2^-hybridized carbon nanostructures and the availability of experimental technologies for the chemical and structural modification of graphene and SWCNTs, it is predicted that an all-carbon graphene/SWCNT hybrid material may become a promising candidate for the creation of phototransistors with tunable bandwidth. In particular, a high-performance photodetector based on graphene/SWCNT van der Waals heterostructures obtained by transferring the CVD-grown graphene onto an ultrathin layer of SWNTs formed on a SiO_2_/Si substrate has already been implemented in practice. The created device exhibited a photoconductive gain of 10^5^ and a fast response time (~100 μs), which indicates the potential use of the graphene/SWCNT composite for light harvesting applications [16]. Liu et al. demonstrated the operation of a large-area photodetector based on hybrid graphene/SWCNT films deposited on a polyethylene terephthalate (PET) substrate. This photodetector showed a high photoresponsivity (~51 A/W) and a fast response time (~40 ms) in the visible wavelength range, as well as excellent mechanical flexibility, good folding strength under severe deformation and bending conditions [22]. The combination of graphene and SWCNTs has potential applications in the development of solar cells, since the graphene/SWCNT hybrid material can combine the high transparency of graphene and the high electrical conductivity of SWCNTs. In particular, Terrones et al. developed a solid-phase pyrolysis method that allows one to synthesize the conductive and transparent composite graphene/CNT films that exhibit increased photoelectric conversion efficiency as compared with devices based on graphene sheets or CNT membranes [12]. The synthesized graphene/CNT composite films were used to assemble solar cells with a C/Si heterojunction which reached a power conversion efficiency of up to 8.5%. Maarouf et al. demonstrated that a hybrid material formed from conductive SWCNTs located on the surface of a graphene sheet has significantly lower sheet resistance as compared with pure graphene at a slight decrease in transparency [23]. Based on the experimental and simulation results, scientists predict that the graphene–SWCNTs hybrid system has high potential for use as a transparent electrode in photovoltaic applications. In addition, a broadband Schottky photodiode array with improved photoresponse has been developed based on the graphene/semiconducting SWCNT film [24]. A graphene-semiconducting SWCNTs thin-film photodetector based on a double-layer stacked heterostructure was fabricated by Cao et al. [25]. This device demonstrates the possibility of photodetection with a high sensitivity of 78 A/W in the visible region of the spectrum.

Nevertheless, studies of the photoelectric properties of graphene/SWCNT composite films with covalent bonding of carbon components are currently fragmentary and need further deepening. In this paper, using in silico methods, we predict the electroconductive and photovoltaic properties of composite films based on two graphene monolayers and SWCNTs located irregularly relative to each other between the layers and covalently bonded to them.

## 2. Materials and Methods

To find supercells of graphene/SWCNTs films with irregular arrangement of nanotubes between graphene monolayers we used the original “magnifying glass method” that was earlier successfully applied for search of supercells of graphene/SWCNTs films with regular arrangement of nanotube graphene monolayers [26]. The “magnifying glass method” includes 3 main stages. In the first stage, an atomistic model of a large finite fragment of a composite film containing 10 or more SWCNTs was built. The equilibrium atomic configuration of the film fragment was found as the result of minimizing the total energy of the structure by the method of molecular dynamics with application of adaptive intermolecular reactive empirical bond-order (AIREBO) potential [27]. The AIREBO potential is an empirical potential that allows modeling of the intermolecular interactions. This potential is based on the empirical Brenner potential, which describes the direct interaction of atoms through covalent bonds. In addition, the AIREBO potential includes the Lennard–Jones interaction and the torsion interaction, which depends on the dihedral angles formed by atoms in a molecule. This allows one to take into account interatomic interactions at distances exceeding the length of covalent bonds, which improves the quality of molecular modeling. Since a fragment of the film atomic structure can contain from 4000 to 6000 atoms depending on the diameter of the SWCNTs and the distance between them, an empirical model was used. At the second stage, the region containing two nanotubes was extracted from a large fragment of the film. To exclude the influence of edge effects, this region was “cut out” from the middle part of the large fragment. The cut-out region represented an expanded supercell of the composite film, for which two translation vectors, L_x_ and L_y_, in the direction of the X (perpendicular to the SWCNT) and Y (along the SWCNT) axes were introduced, respectively. Optimization of the extended supercell structure geometry in this case was performed by the quantum method. Herewith, the lengths of the translation vectors L_x_ and L_y_, as well as coordinates of atoms, were optimized. Due to the polyatomic nature of the resulting supercells (400–800 atoms), we applied the self-consistent charge density functional tight-binding (SCC DFTB) method [28], which, unlike ab initio methods, allowed us to calculate structures containing up to several thousand atoms. At the final stage, the middle part was again cut out from the optimized expanded supercell. This part was a minimal supercell that completely reproducing the structure of the composite film using translation vectors.

## 3. Results and Discussion

### 3.1. Topological Models of Supercells of Graphene/SWCNTComposite Films

**Figure 1:**
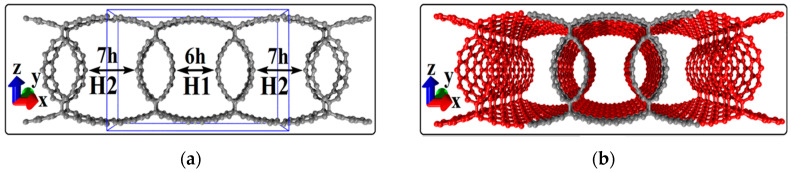
Topological models of graphene/single-wall carbon nanotube (SWCNT) composite films based on SWCNTs (10,0): (**a**) extended supercell of the composite film; (**b**) fragment of the composite film. The atoms of the supercell are highlighted in gray; the fragment of the composite film obtained by translating a supercell along the X and Y axes is highlighted in red.

Using the “magnifying glass method”, we built topological models of supercell of graphene/SWCNT composite films with tubes (m,0), where m = 10, 12, 14, 16. Non-chiral zigzag-type SWCNTs were selected since it was previously shown that only tubes of this topological type can form energy-stable covalent bonds with graphene monolayers [26]. The diameter of the used tubes (0.8–1.3 nm) corresponded to the most frequently synthesized SWCNTs [29,30]. The distance H1 between the SWCNTs in the composite films varied within 0.85–1.21 nm, and the distance H2 within 1.10–2.42 nm. Thus, the distances H1 and H2 varied in the range of 6–14 hexagons. Figure 1a shows an example of a built supercell for the composite film with SWCNTs (10,0) and the distances between neighboring tubes of 6 (H1) and 7 hexagons (H2) (the supercell is highlighted with a blue box). It can be seen that graphene monolayers of the composite have become curvilinear due to bonds with tubes. The fragment of the graphene/SWCNT (10,0) composite atomic structure reproduced by translation of the supercell in the X and Y directions is shown in Figure 1b (gray indicates the initial supercell; red indicates the translated supercells). For each of the built supercells, the energy stability was estimated by the change in the total energy of the studied system according to the following formula:

ΔE = (E_c_ − E_gr_ − E_tube_)/N_atom_ (1)

where E_c_—energy of composite graphene/SWCNT, E_gr_—total energy of graphene monolayers in a composite, E_tube_—total energy of nanotubes in a composite, and N_atom_—number of the composite’s atoms. If the value of ∆E was negative, such a topological configuration of the supercell was considered energetically favorable.

**Figure 2:**
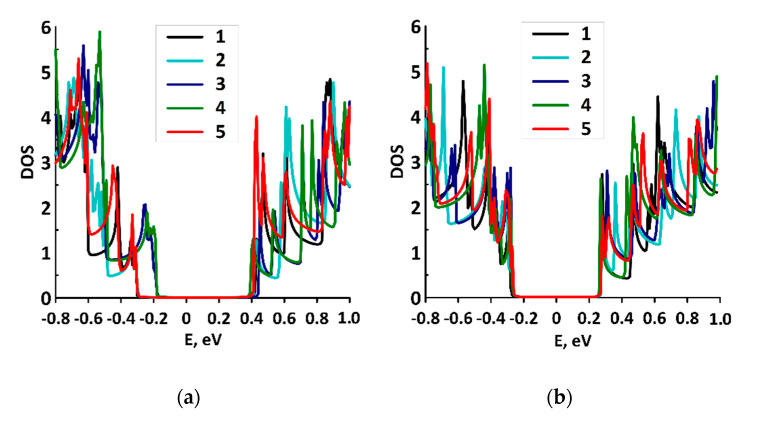

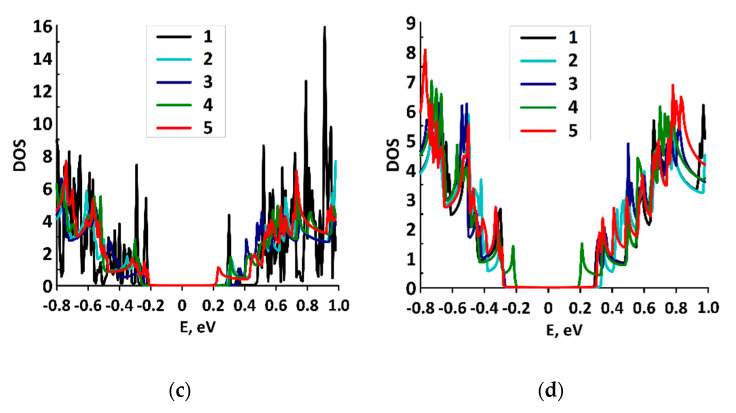
DOS graphs of graphene/SWCNT composite films with different SWCNTs at different distances H1/H2 between SWCNTs: (**a**) film with SWCNT (10,0), numbers 1–5 correspond to the distances H1/H2 6/7–6/11 hexagons; (**b**) film with SWCNTs (12,0), numbers 1–5 correspond to the distances H1/H2 7/8–7/12 hexagons; (**c**) film with SWCNT (14,0), numbers 1–5 correspond to the distances H1/H2 8/9–8/13 hexagons; (**d**) film with SWCNT (16,0), numbers 1–5 correspond to the distances H1/H2 9/10–9/14 hexagons.

Table 1 shows the geometric and energy characteristics of energetically favorable supercells of graphene/SWCNT composite films at the different diameters of SWCNTs and distances between them H1/H2: translation vectors L_x_ and L_y_, film thickness h, changes in total energy ∆E, Fermi level E_f_, and energy gap E_gap_ of the band structure. According to the data in Table 1, the minimum permissible values of the distances H1 and H2 for energetically stable supercells of graphene/SWCNT composite films are determined by the SWCNT diameter. The smaller the diameter, the smaller the minimum values of H1 and H2. The thickness of the films h was determined by the diameter of the SWCNTs and the degree of graphene bending between the tubes. In general, the thickness h = 1.63–2.42 nm. The analysis of the electronic structure of the considered graphene/SWCNT composite films was performed on the basis of the calculated density of electronic states (DOS) distributions. The calculation results showed that all constructed atomistic models of composite films were characterized by the presence of the energy gap of 0.43–0.73 eV. Figure 2a–d show DOS distributions for different topological configurations of graphene/SWCNT composite films. The Fermi level in different configurations ranged from −4.63 eV to −4.7 eV.

### 3.2. Electrical Properties of Graphene/SWCNT Composite Films

**Figure 3:**
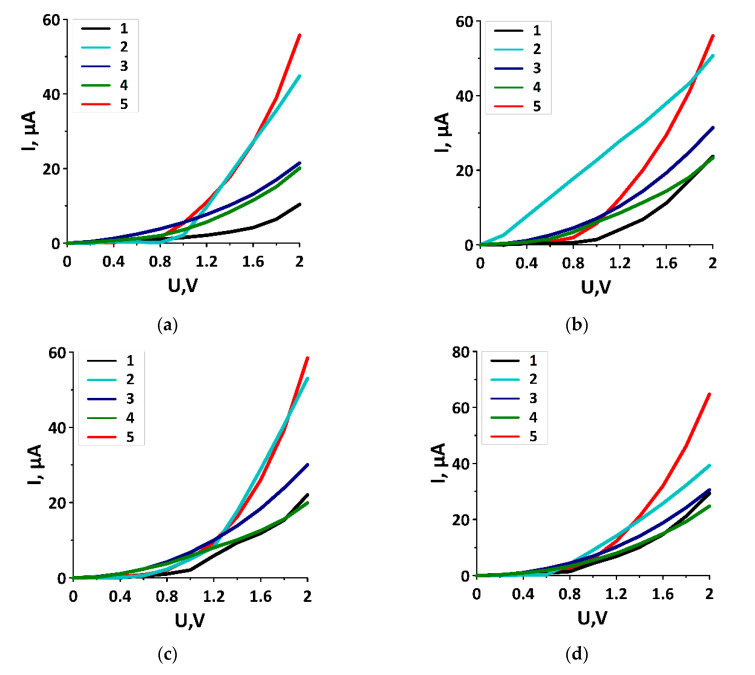
Families of volt-ampere characteristic (VAC) curves of graphene/SWCNT composite films: (**a**) film with SWCNT (10,0) and H1/H2 6/9 hexagons; (**b**) film with SWCNT (12,0) and H1/H2 7/10 hexagons; (**c**) a film with SWCNT (14,0) and H1/H2 8/9 hexagons; (**d**) film with SWCNT (16,0) and H1/H2 9/14 hexagons. The curve with the number “1” denotes the composite film with the corresponding SWCNTs, the curve with the number “2” denotes the SWCNTs from the composite film, the curve with the number “3” denotes the initial SWCNTs, the curve with the number “4” denotes the initial graphene, and the curve with the number “5” denotes the graphene from the composite film.

Further, in order to identify patterns of current transfer, the volt-ampere characteristics (VAC) were calculated for all the considered models of graphene/SWCNT composite films. For the calculation of current transfer in the structure, the Landauer–Butticker formula was used [31]:

(2) $$I=\frac{e}{h}\overset{\infty }{\underset{-\infty }{\int }}T\left(E\right)dE\left[f_{1}\left(E\right)-f_{2}\left(E\right)\right]$$

where *T*(*E*)—transmission function that determines the total quantum mechanical transparency of the conducting structure over all independent conduction channels for an electron with energy *E*; *f*_1_ and *f*_2_—Fermi–Dirac functions that characterize the energy levels of the source and drain corresponding to the energy level of the conducting structure. The VAC was calculated for the direction of current transport along the SWCNTs, since there is no current in the perpendicular direction. Figure 3 shows the families of VAC curves of graphene/SWCNT composite films with different SWCNTs at different distances H1/H2 between SWCNTs at the voltage of 2V (the voltage step is 0.2 V): (a) the film with SWCNT (10,0)—6/9 hexagons; (b) the film with SWCNT (12,0)—7/10 hexagons; (c) the film with SWCNT (14,0)—8/9 hexagons; and (d) the film with SWCNT (16,0)—9/14 hexagons. Table 2 shows the calculated values of the minimum and maximum current for the different film models at the same voltage of 2 V. To assess the influence of the composite film components on the nonlinear nature of current transmission, the VAC of each composite film component was calculated. In Figure 3, the curve with the number “1” denotes the composite film with the corresponding SWCNTs, the curve with the number “2” denotes the SWCNTs from the composite film, the curve with the number “3” denotes the initial SWCNTs, the curve with the number “4” denotes the initial graphene, and the curve with the number “5” denotes the graphene from the composite film.

Figure 3 well illustrates the nonlinear character of current similar to the classical silicon diode. The non-linearity of all VAC graphs of graphene/SWCNT composite films is caused by the presence of the energy gap in the band structure of the films, as shown above. Especially, it is necessary to note the film topological model with SWCNT (12,0). The VAC of an individual tube (12,0) is a straight line in the current–voltage coordinates (see Figure 3b) that is predetermined by the metallic type of conductivity of this tube. However, the composite film based on SWCNT (12,0) demonstrated the VAC of a typical semiconductor. Indeed, as noted above, the formation of covalent bonds between SWCNTs and graphene lead to changes in the conducting properties of both graphene and nanotubes. As a result, the band structure of the composite film exhibits a band gap between the valence and the conduction bands. This effect is typical for all the considered composite film topological models. Analysis of the graphs in Figure 3 also showed that the VAC for different composite films were approximately similar to each other, regardless of the distance between the SWCNTs. Thus, we can conclude that the step of the SWCNT arrangement and the irregularity of their spacing relative to each other did not significantly affect the character of the composite film VAC. In all cases, the current appeared at the voltages of ~0.5–0.6 V according to the value of the band gap. Therefore, the irregular arrangement of nanotubes at the distance between them within 0.9–2.5 nm did not significantly affected the conducting properties of the composite films.

### 3.3. Dynamic Conductivity and Photocurrent of Graphene/SWCNT Composite Films

To study the photovoltaic properties of composite films, it is necessary to know, first of all, the spectrum of the absorption coefficient a(ω). For this purpose, we applied quasi-classical approach, in which the formula for the absorption coefficient and the formulas for the reflection and transmission coefficients were determined based on the classical theory of electrodynamics. To calculate the dynamic conductivity, the quantum theory of irreversible Kubo processes was used. Previously, authors developed the formula for calculating the reflection coefficient of thin films whose thickness was much less than the wavelength of incident radiation [32]. In our case, the film thickness was 1.6–2 nm, which was much less than the wavelengths of the studied range of 200–2000 nm; thus, the formula could be applied without any additional limits:

a(ω) = 1 − |R(ω)|^2^ − |T(ω)|^2^ (3)

where R(ω) = σ_αβ_(ω)Z_0_/2 + σ_αβ_(ω)Z_0_/2—the reflection coefficient, T(ω) = 2/2 + σ_αβ_(ω)Z_0_/2—transmission coefficient, Z_0_—wave resistance, and σ_αβ_(ω)—the optical conductivity tensor. The value Z_0_ was calculated by the formula Z_0_ = E_x_/H_y_ = ηcosθ—for waves of S-polarization (the electric field vector E was perpendicular to the plane of the wave incidence) and Z_0_ = E_x_/H_y_ = η/cosθ—for waves of P-polarization (the vector E lied in the plane of the wave incidence, and the vector of the magnetic field H was perpendicular to it). Coefficient η = 120π Ohm expressed the impedance of the vacuum. Note that sunlight is not polarized, so we calculated the average value of a(ω) for S- and P- polarization waves. The incidence of light waves on the film was considered as normal, i.e., θ = 90°. The complex optical conductivity tensor was calculated by the Kubo–Greenwood formula [33].

**Figure 4:**
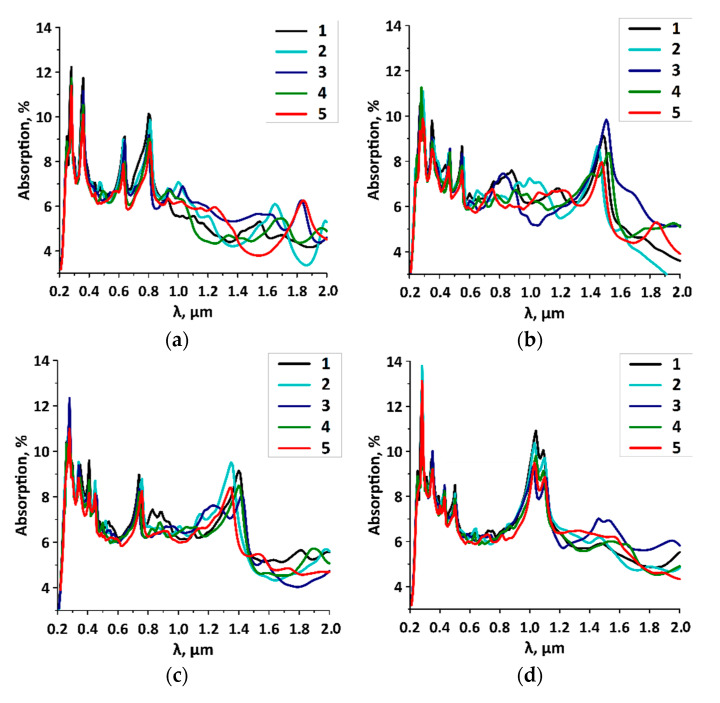

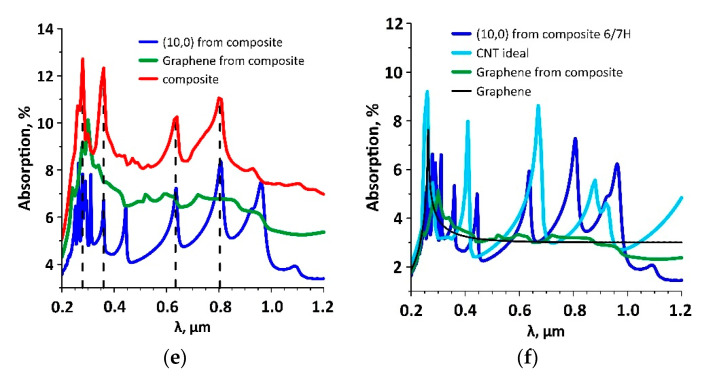
Absorption spectra of graphene/SWCNT composite films with different SWCNTs and distances H1/H2 between them: (**a**) film with SWCNTs (10,0), numbers 1–5 correspond to H1/H2 of 6/7–6/11 hexagons; (**b**) film with SWCNTs (12,0), numbers 1–5 correspond to H1/H2 of 7/8–7/12 hexagons; (**c**) film with SWCNTs (14,0), numbers 1–5 correspond to H1/H2 of 8/9–8/13 hexagons; (**d**) film with SWCNTs (16,0), numbers 1–5 correspond to H1/H2 of 9/10–9/14 hexagons; (**e**) film with SWCNTs (10,0) and H1/H2 6/7 hexagons film components; (**f**) graphene and SWCNT in the composite film with SWCNTs (10,0) and H1/H2 6/7 hexagons, ideal graphene and SWCNT.

The calculated absorption spectra for different variations of the distances between the SWCNTs are shown in Figure 4. For SWCNT (10,0; Figure 4a), SWCNT (12,0; Figure 4b), SWCNT (14,0; Figure 4c), and SWCNT (16,0; Figure 4d), two cases of the distances H1/H2 are shown. These two cases were chosen because of the greatest difference in the absorption spectra. The graphs show that for all types of films, regardless of the diameter of the tubes and the distance between them, there was a noticeable peak of absorption intensity of 11–14% at the wavelength of 265 nm. The presence of this peak in the spectrum is explained by the presence of the graphene sheet, which is known to have an absorption peak of ~7% at the wavelength of 265 nm [34]. The second intensity peak, when a(ω) reaches ~10%, was observed in the IR region at the wavelengths of 1000–1500 nm. It appeared in the spectra of composites with the SWCNTs with diameter of 1 nm or more, regardless of the tubes’ step of arrangement. Another distinctive feature of all a(ω) spectra was the presence of clearly defined absorption peaks in the visible region of 500–750 nm. The position of peaks and their intensity was determined by the type of SWCNT. The distance between the tubes and the irregularity of their location did not have any effect on the position of the peaks in the UV range, but affected the intensity of the absorption peaks. The general trend for all the studied film models was the tendency to decrease the intensity of peaks with an increase in the H1/H2 ratio. In other words, the models with a more noticeable irregular arrangement of tubes had lower absorption in the UV region. A similar trend was observed in the IR range for absorption intensity peaks at wavelengths of 1000–1500 nm. In addition, the peaks themselves slightly changed their position depending on the diameter of the tube. There were no regularities in the distribution of absorption intensity between the intensity peaks in the UV and IR ranges.

To explain the appearance of peaks at certain wavelengths, we calculated the absorption spectra of films, which are only irregularly arranged nanotubes, and the spectra of films made of curvilinear graphene. Figure 4f shows three absorption spectra: for a graphene/SWCNT composite film with tubes (10,0) and a distance between neighboring tubes of 6/7 hexagons (denoted in red), for a graphene/SWCNT film with tubes (10,0) and a spacing step of 6/7 hexagons (denoted in blue), and for curvilinear graphene film (denoted in green). It should be noted that the atomic structures of curvilinear graphene and the deformed nanotube were taken from the supercell of the composite without reoptimization. The calculation results for the graphene/SWCNT composite film with curvilinear graphene are multiplied by 2 to take into account the absorption of both graphene layers. Analysis of the spectral profiles shows that there are two bright peaks in the UV region and in the blue region of the visible range, which are caused by graphene and peaks close to each other of the absorption spectra of nanotubes. These peaks are marked by vertical straight lines in Figure 4e. The other two bright peaks (also marked by vertical straight lines in Figure 4e) are due to the absorption spectra of the nanotube film. The origin of all the aforementioned absorption peaks is determined by the nature of the nanotube itself. To understand this fact, Figure 4f shows the absorption spectra of composite films with deformed SWCNTs (denoted in dark blue) and composite films with ideal SWCNTs (denoted in blue) at the same irregular distance between neighboring tubes. It can be seen that the peaks of the SWCNT spectrum intensity are shifted to the UV region during the formation of a composite accompanied by deformation of nanotubes. In this case, the spectrum of curvilinear graphene (green curve) undergoes insignificant changes in comparison with pristine graphene (black curve).

The spectrum of the photocurrent maximum value and the integral value of the photocurrent were calculated on the basis of absorption spectra. The photocurrent spectrum was calculated by the following formula:

I_max_(ω) = eP_in_∙a(ω)/hω (4)

where P_in_—the power of incident solar radiation, e—electron charge, and hω is the energy of a quantum of solar radiation. Formula (4) allows one to obtain the maximum photocurrent spectrum, since it does not take into account a partial electron–hole recombination. Thus, Formula (4) is applicable for the case of an internal quantum efficiency of 100%, when each absorbed photon generates an electron. Further, on the basis of calculated spectrum, the integral value of the photocurrent was calculated as an integral over the entire considered frequency range. It should be noted that the power of solar radiation is unevenly distributed over the wavelengths. The peak of the maximum intensity of solar radiation is located in the visible light region; then, the intensity decreases exponentially, reaching a minimum in the infrared region [35].

**Figure 5:**
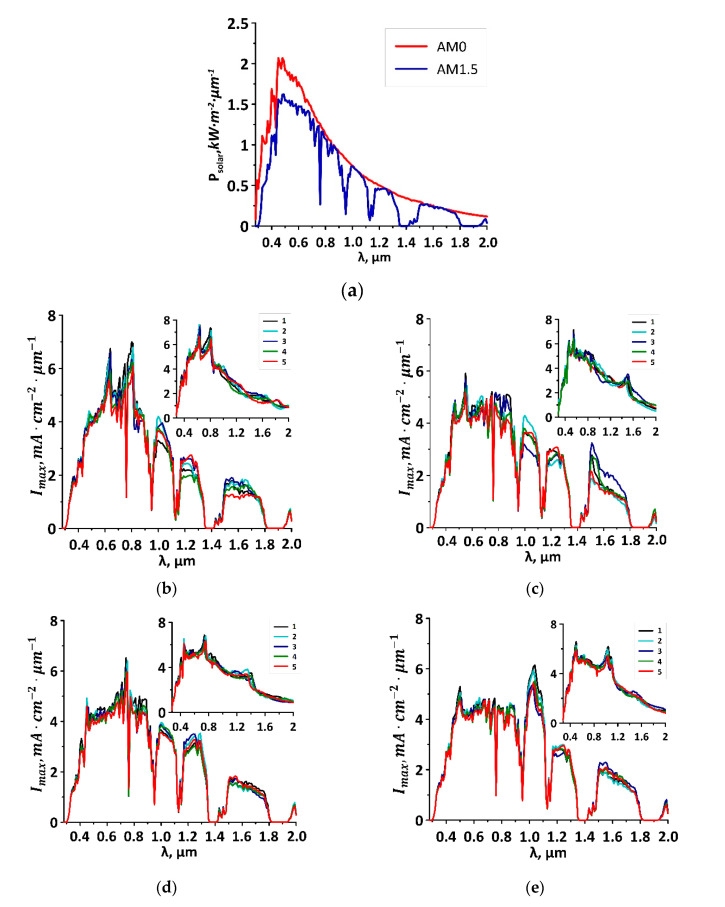
Photocurrent of graphene/SWCNT composite films for the solar spectrum at AM1.5, and in the inserts at AM0: (**a**) solar power spectrum; (**b**) films with SWCNTs (10,0), numbers 1–5 correspond to H1/H2 of 6/7–6/11 hexagons; (**c**) films with SWCNTs (12,0), numbers 1–5 correspond to H1/H2 of 7/8–7/12 hexagons; (**d**) films with SWCNTs (14,0), numbers 1–5 correspond to H1/H2 of 8/9–8/13 hexagons; (**e**) films with SWCNTs (16,0), numbers 1–5 correspond to H1/H2 of 9/10–9/14 hexagons.

The spectra of the photocurrent with the distribution of solar radiation power over wavelengths on the earth’s surface (AM1.5) and outside the atmosphere (AM0) (in inserts) [36] are illustrated in Figure 5 for four composite film topological models with SWCNTs (10,0), (12,0), (14,0), and (16,0). The distances H1/H2 were taken similarly to the cases shown in Figure 4. It can be seen that as the diameter of composite film tubes changed, the spectral peaks shifted, and the H1/H2 ratio affected the amount of photocurrent. For the case of the film with the SWCNTs (10,0), the solar spectrum at AM1.5 demonstrates two distinct peaks at the wavelengths of 650 and 810 nm with a maximum values of 6.9 and 7.1 mA∙cm^−2^, respectively (see Figure 5b). These peaks corresponded to the absorption maxima in Figure 4a. Films with the SWCNTs (12,0) are characterized by only one peak in the visible range at the wavelength of 560 nm with a value of 5.95 mA∙cm^−2^ (see Figure 5c). With an increase in the SWCNT diameter, the value of the photocurrent for waves of visible range decreased to 4–4.5 mA∙cm^−2^. The reason for this is the low absorption intensity in this range, as noted above and shown in Figure 4c,d. Films with the SWNTs (14,0) and (16,0) were characterized by peaks in the IR range (see Figure 5d,e). Film with the SWCNTs (16,0) that demonstrated a clear double absorption peak at the wavelength of 1000–1100 nm also demonstrates the maximum photocurrent of 6.25 mA∙cm^−2^ at the same wavelength range. The film with the SWCNTs (14,0) has an absorption peak at the wavelength of ~1500 nm (see Figure 4c). However, the solar spectrum is characterized by a very low power in this part of the IR range, so there are no large current values that were observed in the photocurrent spectrum of the film with the SWCNTs (14,0). Note that all the described features of the photocurrent spectra are also valid for the case of the solar spectrum AM0.

To determine the efficiency of the considered composite films as materials for photovoltaic applications, it is necessary to identify the integral value of the photocurrent. Table 3 shows the integral values of the photocurrent I_sum_ calculated for the range 0.2–2 µm for all film topological models with different distances between tubes H1/H2. The values of the photocurrent for the visible range of the solar spectrum of 380–780 nm and the integral value of the photocurrent for the IR range of 780–2000 nm are also given.

The analysis of the obtained data presented in Table 3 and in Figure 5 shows that the film with SWCNTs (16,0) at the distances H1/H2 equaled to 9/10 and 9/11 is the most effective both on the earth’s surface and outside the atmosphere. The integral current for the 200–2000 nm range has the maximum value. However, for the visible range (380–780 nm), the maximum photocurrent is observed for the films with SWCNTs (10,0) with distances H1/H2 equaled to 9/10 and 9/11 both on the earth’s surface and outside the atmosphere. According to simulation results, the values of the integral photocurrent outside the atmosphere for considered graphene/SWCNT composite films exceed the calculated values of the photocurrent of some representatives of transition metal dichalcogenides, in particular, MoS_2_ (3.9 mA/cm^2^), MoSe_2_ (4.6 mA/cm^2^) and WS_2_ (2.3 mA/cm^2^) [37]. Based on the obtained results of calculations of the photocurrent, it is predicted that the studied graphene/SWCNT composite films can find application in photovoltaics as a material for creating electrodes. However, further experimental research is needed to develop this topic.

## 4. Conclusions

Thus, we have shown that all the topological models of graphene/SWCNT composite films considered in this paper are semiconductors with an energy gap in the range of 0.43–0.73 eV, which is an important condition for the potential use of such films in photovoltaic applications. According to the simulation results, the electric current flowing through the nanotubes of graphene/SWCNT composite films can reach values of ~50–65 µA at a voltage of 2 V. In this case, the current value is affected by the regularity of the nanotube locations relative to each other and the distance between them. This fact indicates the possibility of topological controlling the electrophysical properties of the considered graphene/SWCNT composite films. Thus, for example, the change in the distance between the SWNTs by just one hexagon leads to a change in the current by 8–17%. Thus, by regulating the distance between the SWCNTs, one can change the behavior of the current–voltage characteristics and the current value. Controlling the nanotube diameter allows us to control the profile of the absorption spectrum of graphene/SWCNT composite films by shifting the maximum peaks. Controlling the distance between the SWCNTs and the regularity of their location allows us to control the absorption intensity without significantly shift of the peaks. It was found that the integral photocurrent for all the considered graphene/SWCNT composite films with an irregular arrangement of SWCNTs is practically independent of the nanotube diameter and the distance between them, which is important in terms of the potential practical application of such films, since it is practically impossible to achieve the same CNT diameters and the distance between them in a real device.

## Figures and Tables

**Table 1:** Geometric and energy characteristics of supercells of graphene/SWCNT composite films.

Parameters	H1/H2	L_x_, Å	L_y_, Å	h, nm	ΔH_f_, kcal/mol∙atom	E_f_, eV	E_gap_, eV
(10,0)	6/7 6/8 6/9 6/10 6/11	32.07 34.46 36.79 39.38 41.72	4.28 4.28 4.29 4.28 4.28	1.645 1.633 1.641 1.634 1.627	−0.23 −0.35 −0.27 −0.22 −0.32	−4.63 −4.63 −4.66 −4.67 −4.63	0.73 0.69 0.6 0.56 0.69
(12,0)	7/8 7/9 7/10 7/11 7/12	36.97 39.33 41.84 44.23 46.67	4.28 4.29 4.28 4.28 4.29	1.897 1.897 1.881 1.884 1.881	−0.19 −0.16 −0.24 −0.19 −0.13	−4.69 −4.69 −4.69 −4.69 −4.69	0.53 0.53 0.52 0.51 0.51
(14,0)	8/9 8/10 8/11 8/12 8/13	41.75 44.28 46.68 49.12 51.59	4.28 4.27 4.28 4.28 4.27	2.167 2.146 2.144 2.120 2.132	−0.22 −0.16 −0.18 −0.21 −0.15	−4.64 −4.66 −4.63 −4.67 −4.69	0.6 0.53 0.61 0.56 0.43
(16,0)	9/10 9/11 9/12 9/13 9/14	46.75 49.16 51.6 54 56.53	4.28 4.28 4.29 4.28 4.28	2.427 2.380 2.367 2.376 2.357	−0.04 −0.12 −0.07 −0.03 −0.10	−4.69 −4.67 −4.68 −4.70 −4.68	0.57 0.62 0.58 0.43 0.59

**Table 2:** Maximum current for graphene/SWCNT composite films and for individual graphene and SWCNT at a voltage of 2V.

Structure Type	(10,0)	(12,0)	(14,0)	(16,0)
Graphene/SWCNT Composite Film	55.73 µA	56.02 µA	58.45 µA	64.72 µA
Ideal Graphene	21.49 µA	34.42 µA	30.06 µA	30.55 µA
Ideal SWCNT	44.81 µA	50.7 µA	53.03 µA	39.24 µA
Graphene in a Composite Film	20.1 µA	23.3 µA	19.89 µA	24.73 µA
SWCNT as Part of a Composite Film	10.38 µA	23.69 µA	22.05 µA	29.3 µA

**Table 3:** Integral value of the photocurrent of composite films.

**AM0**							
I_sum_ for the Emission Spectrum 200–2000 nm,$\;\mathrm{mA}\cdot {\mathrm{cm}}^{-2}$							
(10,0)		(12,0)		(14,0)		(16,0)	
6/7	5.19	7/8	5.48	8/9	5.60	9/10	5.61
6/8	5.22	7/9	5.28	8/10	5.48	9/11	5.57
6/9	5.31	7/10	5.46	8/11	5.39	9/12	5.59
6/10	4.98	7/11	5.31	8/12	5.34	9/13	5.40
6/11	5.01	7/12	5.17	8/13	5.26	9/14	5.39
I_sum_ for the Visible Part of the Solar Spectrum 380–780 nm, $\mathrm{mA}\cdot {\mathrm{cm}}^{-2}$							
(10,0)		(12,0)		(14,0)		(16,0)	
6/7	2.19	7/8	2.08	8/9	2.11	9/10	2.02
6/8	2.10	7/9	2.03	8/10	2.07	9/11	1.98
6/9	2.08	7/10	1.99	8/11	2.04	9/12	1.96
6/10	2.01	7/11	1.96	8/12	2.00	9/13	1.93
6/11	1.96	7/12	1.95	8/13	1.97	9/14	1.92
**AM1.5**							
I_sum_ for the Emission Spectrum 200–2000 nm, $\mathrm{mA}\cdot {\mathrm{cm}}^{-2}$							
(10,0)		(12,0)		(14,0)		(16,0)	
6/7	3.90	7/8	4.03	8/9	4.06	9/10	4.17
6/8	3.96	7/9	3.87	8/10	3.98	9/11	4.13
6/9	3.94	7/10	3.99	8/11	3.94	9/12	4.09
6/10	3.73	7/11	3.87	8/12	3.86	9/13	4.02
6/11	3.72	7/12	3.76	8/13	3.85	9/14	3.99
I_sum_ for the Visible Part of the Solar Spectrum 380–780 nm, $\mathrm{mA}\cdot {\mathrm{cm}}^{-2}$							
(10,0)		(12,0)		(14,0)		(16,0)	
6/7	1.78	7/8	1.68	8/9	1.72	9/10	1.64
6/8	1.71	7/9	1.65	8/10	1.67	9/11	1.60
6/9	1.69	7/10	1.64	8/11	1.65	9/12	1.59
6/10	1.63	7/11	1.59	8/12	1.63	9/13	1.56
6/11	1.60	7/12	1.58	8/13	1.60	9/14	1.55
